# The role of subtalar extrarticular screw arthroereisis (SESA) in surgical treatment of tarsal coalitions

**DOI:** 10.1186/s10195-025-00887-2

**Published:** 2025-10-22

**Authors:** Maurizio De Pellegrin, Lorenzo Marcucci, Nicola Guindani, Lorenzo Brogioni, Dario Fracassetti

**Affiliations:** 1 Pediatric Orthopedic Unit, Piccole Figlie Hospital, Parma, Italy; 2 Department of Orthopedic, ASST Ospedale Papa Giovanni XXIII, Bergamo, Italy; 3 Orthopedic Residency Program, University of Verona, 37134 Verona, Italy

**Keywords:** Symptomatic flatfoot, Rigid flatfoot, Hindfoot valgus, Tarsal coalitions, Subtalar arthroereisis, SESA, Calcaneonavicular coalition, Talocalcaneal coalition

## Abstract

**Background:**

Talocalcaneal (TCC) and calcaneonavicular (CNC) coalitions are the most common cause of rigid symptomatic flatfoot in children. After resection, calcaneal lengthening osteotomy or arthrodesis are usually reported as second step surgery for correction of the most frequent valgus hindfoot deformity. More recently, coalition resection and minimally invasive subtalar extraarticular screw arthroereisis (SESA) for hindfoot valgus correction in one step have been described. We report the functional mid-term results of patients treated in adolescence with resection and valgus correction with SESA.

**Methods:**

Between 2008 and 2024 data were collected from 25 patients (18 M, 7 F) affected by TCC (*n* = 16, 7R, 9L) and CNC (*n* = 16, 8R, 8L), all with symptomatic rigid flatfeet (*n* = 32). Average age at surgery was 12.8 ± 1.4 years (9.8–16.4 years, median 12.8). All patients underwent resection and SESA for correction of residual hindfoot valgus deformity; 31/32 feet had postoperative American Orthopaedic Foot and Ankle Society (AOFAS) Ankle-Hindfoot score. Mann–Whitney test was used for comparison between TCC and CNC outcomes.

**Results:**

Average follow-up (FU) was 4.7 ± 3.2 years (6 months–11.9 years, median 3.7) with a mean age at FU of 17.5 ± 3.3 years (13.2–25.4 years, median 16.8). Overall average AOFAS Ankle-Hindfoot score was 95.6 ± 5.7 and 94.3 ± 6.6 for TCC and 96.7 ± 4.6 for CNC, respectively. Subgroup scores for pain, function, and alignment were 37.3 ± 4.6, 48.7 ± 2.4, and 8.3 ± 2.4 for TCC and 38.1 ± 4.0, 48.6 ± 6.2, and 10.0 for CNC, respectively, showing a statistically significant difference between TCC and CNC only for alignment (*p* = 0.014). No patients had additional surgery for complications or valgus recurrence.

**Conclusions:**

Symptomatic rigid flatfeet affected by TCC and CNC and treated in adolescence with coalition resection and SESA for residual hindfoot valgus correction achieved good to excellent results in all cases. Further surgery to correct malalignment was avoided.

**Level of evidence:**

Level IV, retrospective study.

## Introduction

Tarsal coalitions are a non-common issue in pediatric orthopedics but the most frequent cause of rigid flatfoot [1]. Although the true incidence of tarsal coalitions is unknown, as many cases are not symptomatic, estimates are between 1% and 13% [2, 3] with a bilateral presentation in 50% of cases [4]. The coalition starts as a syndesmosis with fibrous tissue, which undergoes metaplasia to cartilage synchondrosis and subsequently to bone synostosis [5]. Therefore, although the coalition develops during embryogenesis, the synostosis forms only during growth, with symptom onset occurring when the coalition ossifies; this generally is between 12 and 16 years for talocalcaneal (TCC) and between 8 and 12 years for calcaneonavicular (CNC) [6].

All tarsal bones can be involved with TCC and CNC, representing more than 90% of all tarsal coalitions [7].

Relative incidence of TCC and CNC is 37% and 53%, respectively [8]. Type of coalition seems not to be an indicative factor in determining outcomes [9–11]. More recently, a review of 1284 tarsal coalition reported an overall clinical success rate of 79% and 81% and pooled complication rates of 4% and 6% for TCC and CNC treatment, respectively [12].

As the subtalar motion is progressively limited, the foot becomes symptomatic and increasingly flat and rigid. The rotatory and gliding motion of the subtalar joint, if restricted, limits the physiological compensatory external rotation of the foot during gait, forcing the calcaneus in a fixed valgus position with flattening of the arch [6]. Although valgus hindfoot deformity has been addressed in many studies, in some patients the arch develops and the hindfoot may be in neutral or in varus position [13, 14].

Pain represents a main symptom and is reported in the site of coalition and on the lateral submalleolar region due to hindfoot valgus deformity, leading to calcaneal-malleolar impingement [15, 16]. Many authors reported a high percentage of failure after only resection procedures when hindfoot valgus deformity is still present [17–20].

The surgical option to correct hindfoot valgus deformity in adults is usually a subtalar arthrodesis [21, 22].

Other authors reported calcaneal lengthening osteotomy for valgus deformity correction in a different surgical time, at the onset of foot pain [17–19].

The aim of this study is to present the functional mid-term results of adolescents treated with subtalar arthroereisis (SESA) for valgus hindfoot deformity correction after resection of TCC and CNC.

## Material and methods

Between 2008 and 2024 data were collected from 25 patients (18 M, 7 F), 12 affected by TCC (*n* = 16, 7R, 9L) and 13 by CNC (*n* = 16, 8R, 8L), all with rigid symptomatic flatfeet (*n* = 32). Data concerning the first 20/25 patients had already been used and published in a previous work from the same authors [23]; from this study, only part of patients’ data, which underwent a one-step procedure with coalition resection and SESA, were included in the present study. Moreover, 1 of these 20 patients later underwent the same surgical procedure for the contralateral side, was reevaluated and his data are updated in the present study.

Average age at surgery was 12.8 ± 1.4 years (9.8–16.4 years, median 12.8).

Average age of TCC patients was 12.8 ± 1.6 years (range 10.5–16.4, median 12.5).

Average age of CNC patients was 12.8 ± 1.1 years (range 9.8–14.6, median 13.0).

In total, 12 patients (6 M, 6 F) had TCC, 3 bilateral (3 M, 1 F), for a total of 16 feet, and 13 patients (12 M, 1 F) had a CNC, 3 bilateral (3 M), for a total of 16 feet.

For hindfoot alignment clinical evaluation the angle in standing position [24, 25] and the plantar malleoli view sign (PMVS) in lying position were used [23, 26]. The heel valgus angle is considered pathological if greater than 10° [25]. According to the PMVS, an aligned hindfoot should have both the medial and the lateral malleoli visible when seen from a plantar view. When a valgus deformity is present, only the medial malleolus is visible. When a varus deformity is present, only the lateral malleolus is visible [23, 26].

All patients’ feet were evaluated with x-ray in weight bearing. Following further imaging was performed for diagnosis and surgical planning: 18 oblique x-ray projection (7 TCC, 11 CNC), 13 computed tomography (CT) scan (9 TCC, 4 CNC), and 22 magnetic resonance imaging (MRI) scan (11 TCC, 11 CNC) (Tables 1 and 2).

**Table 1:** Data of patients affected by TCC and treated with coalition resection and hindfoot valgus correction with subtalar extraarticular screw arthroereisis (SESA)

Patient	Gender	Side	x-ray (AP. LL)	x-ray (oblique projection)	CT	MR	Age at surgery (years)	Age at FU (years)
1	M	R	X	X	X		12.51	17.14
	M	L	X	X	X		13.43	17.14
2*	M	L	X				12.21	12.71
3	F	L	X		X		11.32	19.28
4	M	L	X	X		X	12.46	24.33
5	F	R	X	X		X	10.92	17.77
6	F	R	X			X	11.89	21.13
	F	L	X			X	12.88	21.13
7	M	L	X	X	X		15.17	18.18
8	M	R	X		X	X	11.59	13.24
	M	L	X		X	X	12.76	13.24
9	F	R	X			X	14.78	16.94
10	M	R	X		X	X	12.56	16.82
	M	L	X		X	X	16.39	16.82
11	F	L	X	X	X	X	13.36	15.59
12	F	R	X	X		X	10.50	14.70

*Further follow-up was not conducted as the patient relocated to another country. Preoperative imaging performed, age at surgery, and age at follow-up are reported. *AP* antero-posterior projection, *LL* latero-lateral projection

**Table 2:** Data of patients affected by CNC and treated with coalition resection and correction of hindfoot valgus with subtalar extraarticular screw arthroereisis (SESA)

Patient	Gender	Side	x-ray (AP. LL)	x-ray (oblique projection)	CT	MR	Age at surgery (years)	Age at FU (years)
1	M	L	X		X		13.53	17.29
2	M	L	X	X	X	X	12.18	16.12
3	M	L	X	X	X	X	13.09	22.30
	M	R	X	X			14.25	22.30
4	M	R	X			X	12.85	16.45
	M	L	X	X			11.42	16.45
5	M	L	X	X			12.28	15.66
	M	R	X			X	13.53	15.66
6	F	R	X			X	9.82	13.39
7	M	L	X			X	11.66	19.06
8	M	R	X	X		X	14.26	25.39
9	M	L	X	X		X	12.95	15.60
10	M	R	X	X		X	13.07	15.04
11	M	L	X	X		X	12.45	15.12
12	M	R	X	X			13.21	13.75
13	M	R	X	X	X	X	14.64	15.40

Preoperative imaging performed, age at surgery, and age at follow-up are reported. *AP* antero-posterior projection, *LL* latero-lateral projection

**Fig. 1:**
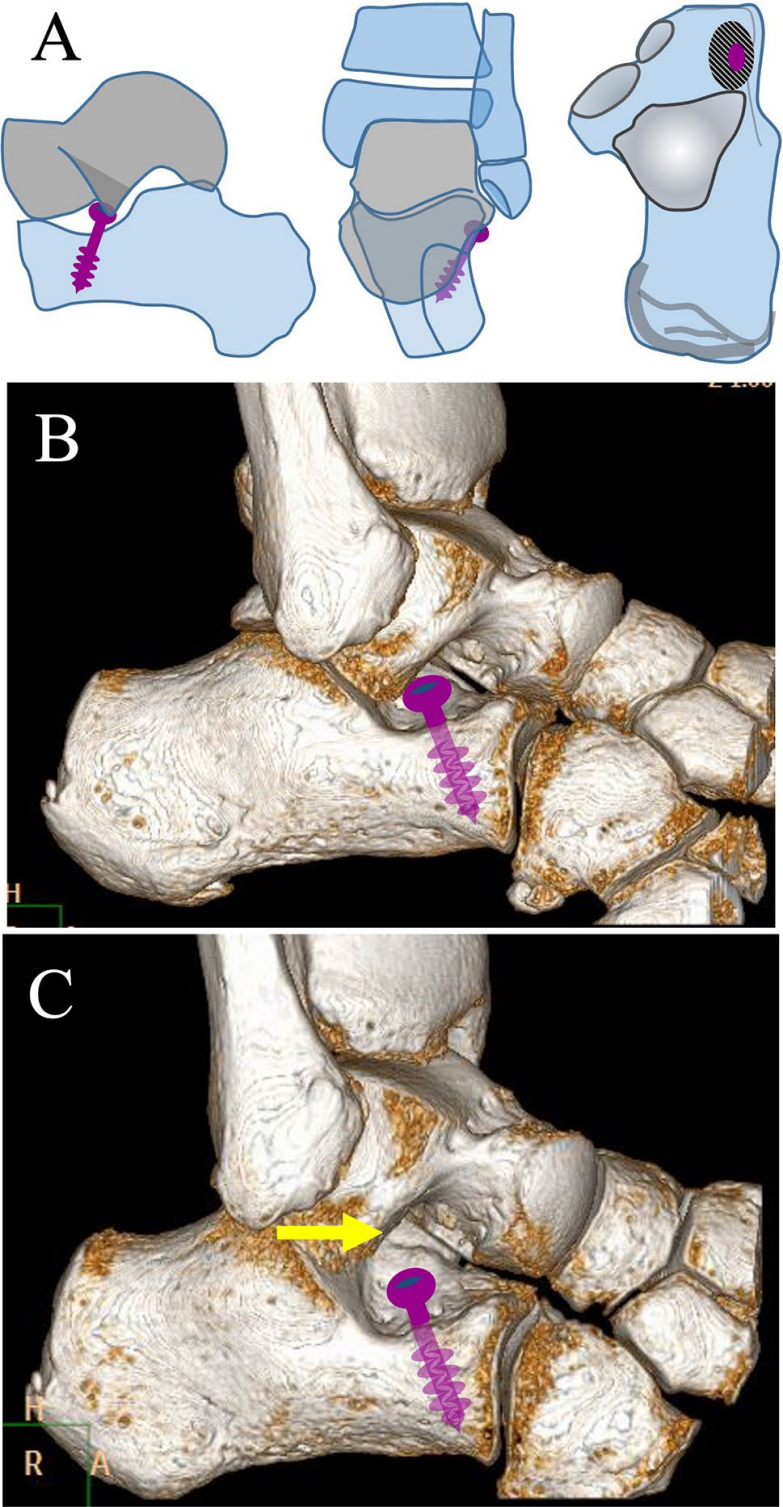
Subtalar arthroereisis with SESA technique. The technique requires a cancellous screw, inserted into the calcaneus at the level of the sinus tarsi, under the talus lateral process. **A** Diagram of the screw position in the calcaneus. **B** Three-dimensional (3D) reconstruction CT scan demonstrating the effect of the screw on the talus lateral process; in pronation the effect of the head of the screw is clearly visible (mechanical block). **C** In supination the screw head is not in contact with the talus (arrow)

All feet (*n* = 32, TCC *n* = 16, CNC *n* = 16) underwent coalition resection and subtalar extraarticular screw arthroereis (SESA) during the same surgical session [26–29] (Fig. 1).

For TCC resection, medial incision was made right under the medial malleolus over the coalition site spanning along the length of the subtalar joint. After identifying the neurovascular bundle, a longitudinal incision was made to open the sheaths of the tibialis posterior (TP) and flexor digitorum longus (FDL) tendons. Moreover, the flexor hallucis longus (FHL) tendon, which is located under the sustentaculum tali and represents a landmark for the above present coalition, was identified. The TP was retracted dorsally and the FDL and FHL plantarly to expose the coalition site [20]. After coalition resection foot mobility and hindfoot alignment using PMVS were clinically evaluated [26]. If residual valgus deformity (positive medial malleolus view sign) was present, SESA was performed through a second skin incision of approximately 1.0 cm length at the sinus tarsi level. The subtalar arthroereisis technique was first described by Alvarez and published by Burutaran in 1979 [30].

**Fig. 2:**
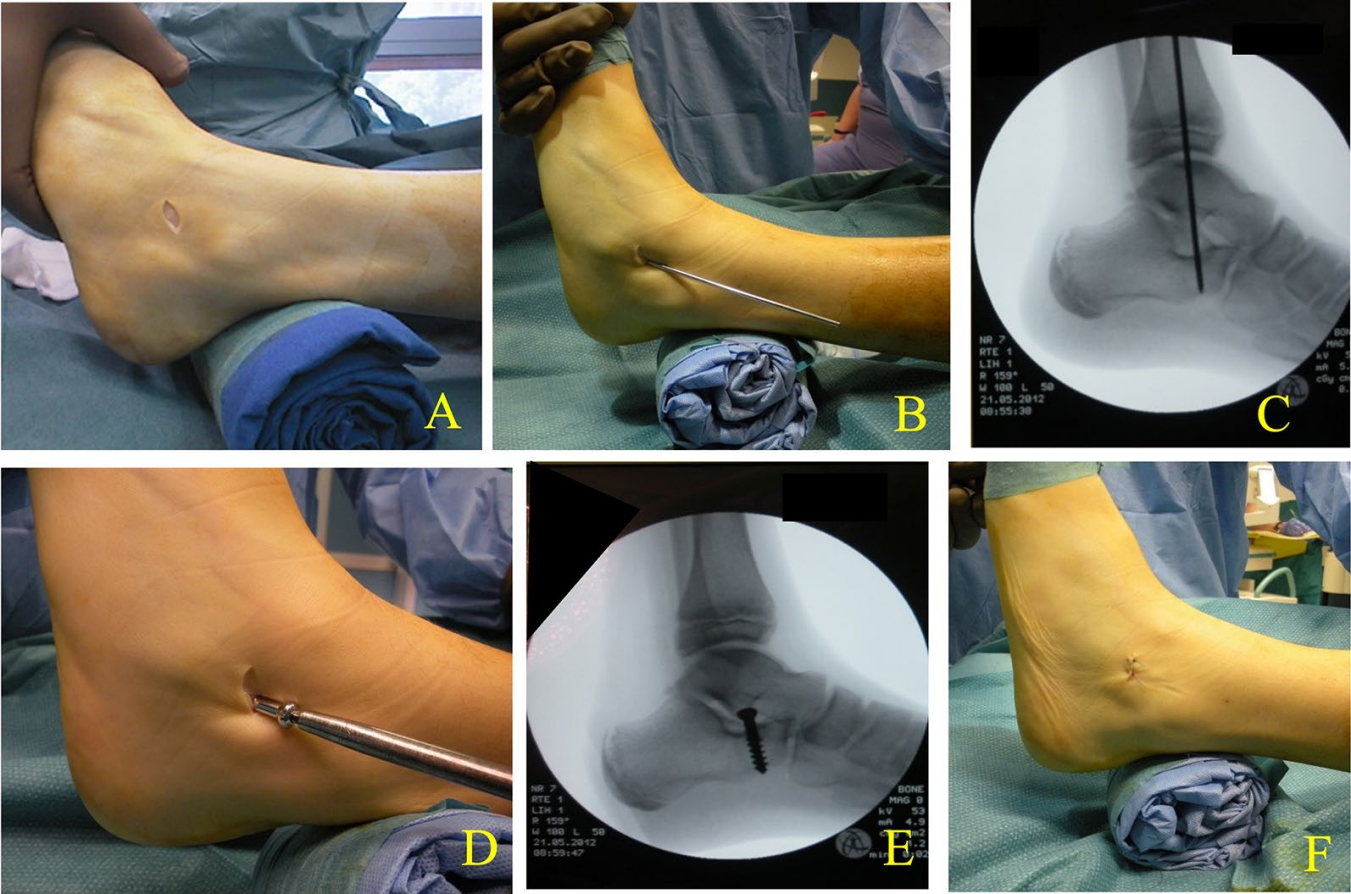
SESA step by step. **A** The 1 cm skin incision at the level of the sinus tarsi. **B** With the foot in neutral position and maximal supination, introduction of a *K*-wire into the calcaneus with an angle of approximately 20° from posterior to anterior and from lateral to medial. **C** x-Ray control to check the correct position of the *K*-wire. **D** The *K*-wire is then removed, and a 3.2 mm reamer is inserted for 2–3 mm to aid the screw entrance. The screw is then inserted until it reaches the position under the talus lateral process. Effectiveness of procedure is tested using the plantar malleoli view sign (PMVS). **E** Intraoperative x-ray shows correct position of the screw. **F** Suture in layers and skin suture

After skin incision, attention must be paid to the sensitive branch of the sural nerve, which may cross the incision. Bluntly, the calcaneus and the floor of the sinus tarsi are reached. The foot is kept by the assistant in maximum supination, which is possible after the previously performed coalition resection. A *K*-wire with a diameter of 2.0 mm is inserted cranio-caudally, from posterior to anterior with an angle of approximately 20°, from lateral to medial, as close as possible anteriorly to the lateral malleolus. The correct position of the *K*-wire is controlled with fluoroscopy. The *K*-wire is then removed and a 3.2 mm reamer is inserted in the same position. The screw used is steel half threaded cancellous screw of 3.0 cm length and with diameter of 4.5/6.5 mm [26]. The screw is inserted into the calcaneus until it reaches a position under the talus lateral process; the thread disappears completely in the calcaneus (Fig. 2). The effectiveness of the stop of the calcaneus against the talus, more anatomical precisely against the plantar surface of the talus lateral process and the valgus correction, is verified by observing the position of the hindfoot with respect to the longitudinal axis of the leg and according to the PMVS. The deep tissues are sutured above the head of the screw; then the subcutaneous tissues and skin are sutured.

For CNC resection, a dorso-lateral incision over the calcaneonavicular region and a longitudinal dissection of the extensor digitorum brevis was performed to expose the coalition site [20]. Once mobility of calcaneonavicular joint was reached after coalition resection, hindfoot axis was intraoperatively evaluated through the PMVS [26]. If residual valgus deformity (positive medial malleolus view sign) was present, SESA was performed enlarging of 2 cm the same surgical incisions to reach the sinus tarsi.

Postoperative care has changed over the years. Until 2020 an immobilization cast was applied for 2–4 weeks, allowing weight bearing. The time of immobilization has decreased over the following years. Postoperative care in the last 5 years was with a cast for 1 week or without cast, only applying an elastic bandage and allowing full weight bearing as soon as possible and as tolerated. The patient is instructed to actively perform flexion–extension exercises of the ankle in the postoperative period. Sport activities are forbidden for 1 month.

All patients were evaluated postoperatively through the AOFAS Ankle-Hindfoot score, divided into three main sections: pain (max 40 points), overall function (max 50 points), and alignment (max 10 points). Function described following seven subgroups: activity limitations (max 10 points), walking distance (max 5 points), walking surface (max 5 points), gait abnormalities (max 8 points), sagittal motion (max 8 points), hindfoot motion (max 6 points), and ankle–foot stability (max 8 points). The maximum possible score is 100 with scores resulting: < 70 = poor, 70–79 = fair, 80–89 = good, 90–100 = excellent [31].

In total, 31/32 feet had postoperative American Orthopaedic Foot and Ankle Society (AOFAS) Ankle-Hindfoot score.

## Statistical analysis

To select the most appropriate statistical test, the assumption of a normal distribution was assessed using the Shapiro–Wilk test, while the assumption of variance equality was verified with the Levene’s test. Subsequently, the Mann–Whitney *U* test for independent nonparametric samples was employed to compare the mean AOFAS test scores across both main groups and subgroups, except for the ankle-hindfoot stability subgroup, where all the patients achieved the maximum score in both TCC and CNC groups. The level of significance was set at *p* < 0.05.

## Results

Preoperative clinical evaluation showed in all patients in weight bearing position valgus hindfoot deformity (hindfoot alignment angle > 10°) and a positive PMVS for valgus deformity. After coalition resection, all feet were intraoperatively evaluated with PMVS: 32 feet (16 TCC, 16 CNC) showed residual hindfoot valgus deformity. After SESA, intraoperatively PMVS showed correct alignment of all feet.

Patients filled in the ankle and hindfoot AOFAS score survey at follow-up (FU). One patient affected by TCC moved to another country, and unfortunately had only 6 months of FU and did not undergo AOFAS questionnaire.

Average FU was 4.7 ± 3.2 years (6 months–11.9 years, median 3.7) with a mean age at FU of 17.5 ± 3.1 years (13.2–25.4 years, median 16.8).

Overall results showed an AOFAS average score of 95.6 ± 5.7 (80–100) and 94.3 ± 6.6 (80–100) for TCC and 96.7 ± 4.6 (86–100) for CNC, respectively.

No patients had additional surgery for complications or valgus recurrence.

Further results are reported in detail separately for TCC and CNC.

### TCC

At preoperative evaluation all patients (12/12) were symptomatic with pain in the site of coalition.

**Fig. 3:**
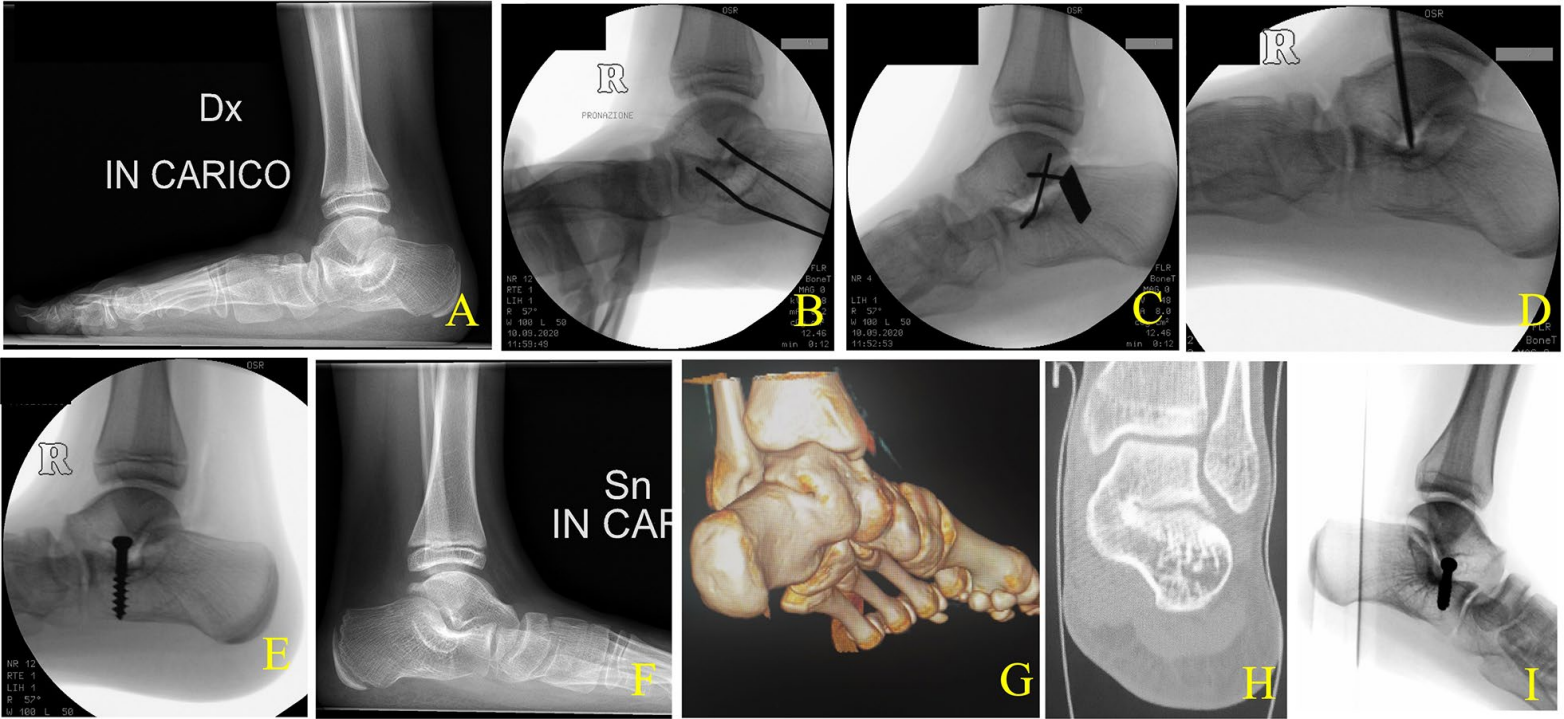
Adolescent affected by bilateral TCC **A** Preoperative weight bearing lateral view x-ray of the right foot with a rigid symptomatic flatfoot deformity in a 12-year-old boy. **B** x-Ray during coalition resection showing the two parallel distractor pins into the talus and calcaneus, respectively. **C** x-Ray after resection showing the achieved mobility of the subtalar joint. See the divergent distractor pins. **D** SESA procedure with the *K*-wire showing the correct point for screw insertion into the calcaneus. **E** Postoperative x-ray after SESA. The correct anatomical talo-calcaneal relationship is maintained. **F** Weight bearing lateral view x-ray of the left foot with a rigid asymptomatic flatfoot deformity of the same patient at 12 years of age. **G** 3D-CT reconstruction at 16 years of age showing a complete osseous coalition of the rigid left foot, which has become symptomatic. **H** CT slice showing the dimension of the coalition. **I** Postoperative x-ray after coalition resection and SESA

Mean age at FU was 17.8 ± 3.0 years (13.2–24.3 years, median 17.1). Postoperative average AOFAS Ankle-Hindfoot score was 94.3 ± 6.6 (80–100). The average score for sections pain, function, and alignment were, respectively: 37.3 ± 4.6, 48.7 ± 2.4, and 8.3 ± 2.4. Table 3 summarized AOFAS outcomes at FU for TCC. One patient did not undergo AOFAS questionnaire. Excellent results were obtained in 13/15 feet and good results in 2/15 (Fig. 3). At FU, two feet were rigid, not showing a recurrence of the hindfoot valgus deformity. No additional surgery was required except in one case for removal of SESA screw 5.6 years after the implant.

**Table 3:** AOFAS evaluation scores at follow-up of patients with TCC after coalition resection and SESA

AOFAS												
Patient	Side	PAIN (max 40 points)	FUNCTION (max 50 points)								ALIGNMENT (max 10 points)	Total
			*Subgroups*									
			Activity limitations, support requirements (max 10 points)	Maximum walking distance, blocks (max 5 points)	Walking surfaces (max 5 points)	Gait abnormality (max 8 points)	Sagittal motion (flexion plus extension) (max 8 points)	Hindfoot motion (inversion plus eversion) (max 6 points)	Ankle-hindfoot stability (anteroposterior, varus-valgus) (max 8 points)	Overall function		
1	R	40	10	5	5	8	8	6	8	50	10	100
	L	30	10	5	5	8	8	6	8	50	10	90
2	NP											
3	L	40	10	5	5	8	8	6	8	50	10	100
4	L	40	10	5	5	8	8	6	8	50	10	100
5	R	40	10	5	5	8	8	6	8	50	10	100
6	R	40	10	5	5	8	8	6	8	50	5	95
	L	40	10	5	5	8	8	6	8	50	5	95
7	L	30	10	5	5	8	8	6	8	50	10	90
8	R	40	10	5	5	8	8	6	8	50	10	100
	L	30	10	5	5	4	8	3	8	43	10	83
9	R	30	10	5	3	8	8	3	8	45	5	80
10	R	40	10	5	5	4	8	6	8	46	5	91
	L	40	10	5	5	4	8	6	8	46	5	91
11	L	40	10	5	5	8	8	6	8	50	10	100
12	R	40	10	5	5	8	8	6	8	50	10	100

*NP not performed; AOFAS score was not completed as the patient relocated to another country. Pain, overall function with subgroups for activity limitations, walking distance, walking surface, gait abnormalities, sagittal motion, hindfoot motion, ankle–foot stability, and alignment are reported

### CNC

At preoperative evaluation all patients (13/13) reported spontaneous pain in the site of coalition and all suffered pain in inversion movements.

**Fig. 4:**
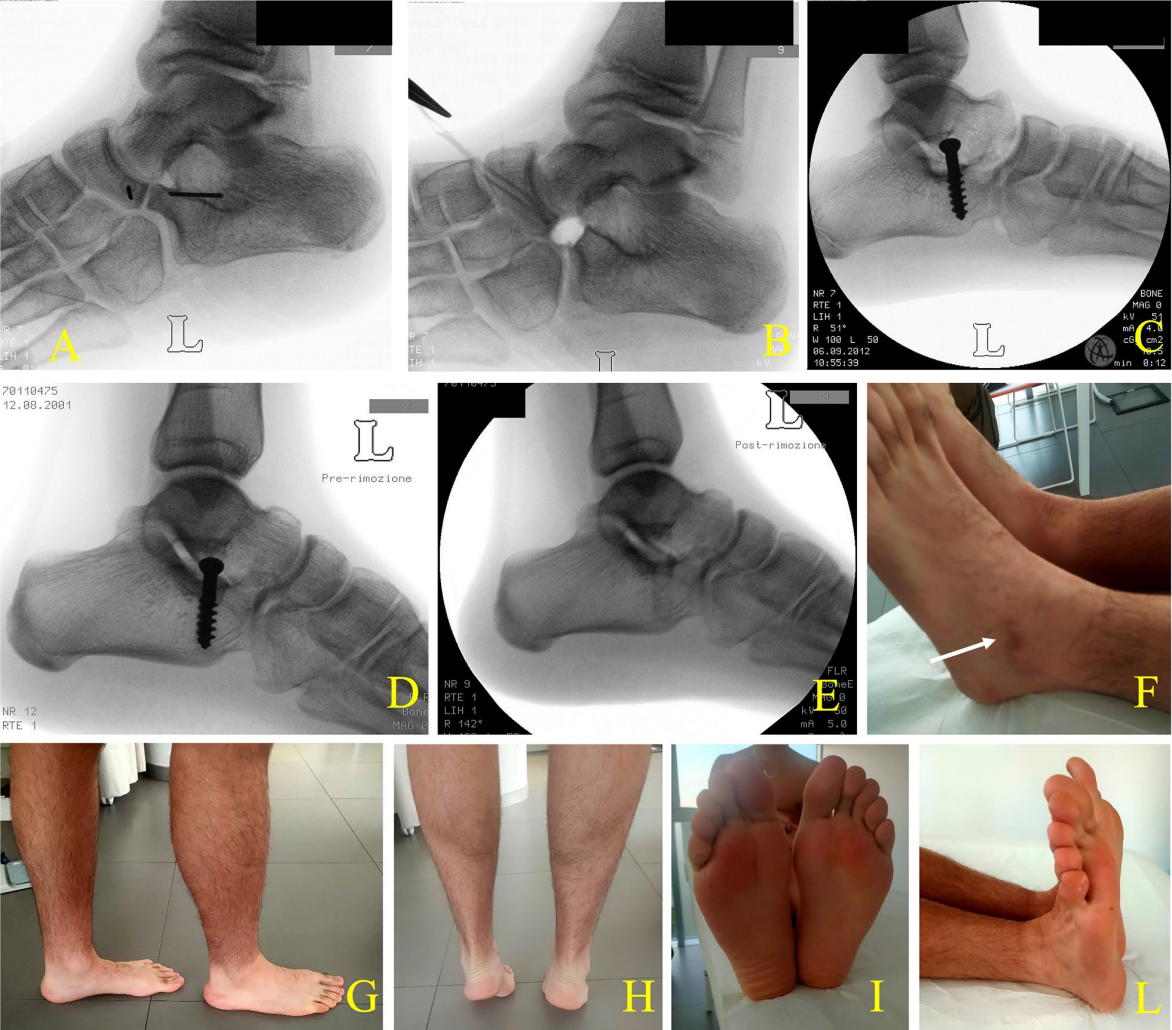
An 11-year-old boy affected by symptomatic rigid flatfoot deformity in CNC of the left foot. **A** Oblique x-ray projection shows the CNC. **B** Intraoperative radiographic evaluation after resection. **C** postoperative x-ray after subtalar arthroereisis with SESA technique for correction of the residual valgus deformity. **D** Preoperative x-ray before screw removal at 16 years and 10 months of age. **E** Postoperative x-ray after screw removal. **F** Skinscar (arrow) at follow-up at 23 years of age. **G** Medial arch at FU. **H** Functional outcome in tiptoes standing with normal pain-free range of motion and hindfoot alignment. **I** Plantar view. **L** Symmetrical ankle extension with right foot for comparison

Mean age at FU was 17.2 ± 3.5 years (13.4–25.4 years, median 15.9). Postoperative average AOFAS Ankle-Hindfoot score was 96.7 ± 4.6 (86–100). Subgroup scores for pain, function, and alignment were 38.1 ± 4.0, 48.6 ± 6.2, and 10.0 ± 0, respectively. Table 4 presented AOFAS outcomes at FU for CNC. Excellent results were obtained in 15/16 feet, and good results in 1/16 (Fig. 4). At FU, two feet were rigid, not showing a recurrence of the hindfoot valgus deformity. One underwent screw removal for pain.

**Table 4:** AOFAS evaluation scores at follow-up of patients with CNC after coalition resection and SESA

AOFAS												
Patient	Side	PAIN (max 40 points)	FUNCTION (max 50 points)								ALIGNMENT (max 10 points)	TOTAL
			*Subgroups*									
			Activity limitations, support requirements (max 10 points)	Maximum walking distance, blocks (max 5 points)	Walking surfaces (max 5 points)	Gait abnormality (max 8 points)	Sagittal motion (flexion plus extension) (max 8 points)	Hindfoot motion (inversion plus eversion) (max 6 points)	Ankle-hindfoot stability (anteroposterior, varus-valgus) (max 8 points)	Overall function		
1	L	40	10	6	5	8	8	3	8	48	10	98
2	R	40	10	5	5	8	8	6	8	50	10	100
3	L	40	10	5	5	8	8	6	8	50	10	100
	R	40	10	5	5	8	8	6	8	50	10	100
4	R	40	10	5	5	8	8	6	8	50	10	100
	L	40	10	5	5	8	8	6	8	50	10	100
5	L	40	10	5	5	8	8	6	8	50	10	100
	R	30	10	5	5	8	8	6	8	50	10	90
6	L	40	10	5	5	8	8	6	8	50	10	100
7	R	40	10	5	5	8	8	6	8	50	10	100
8	L	40	10	5	5	8	8	3	8	47	10	97
9	R	40	10	5	5	8	8	6	8	50	10	100
10	L	30	10	5	5	8	4	6	8	46	10	86
11	L	40	7	5	3	8	6	6	8	43	10	93
12	R	40	10	5	3	4	8	6	8	44	10	94
13	R	30	10	5	5	8	8	6	8	50	10	90

Pain, overall function with subgroups for activity limitations, walking distance, walking surface, gait abnormalities, sagittal motion, hindfoot motion, ankle–foot stability, and alignment are reported

AOFAS score for TCC and CNC was compared using Mann–Whitney test for nonparametric independent groups; overall no statistical significance (*p*-value < 0.05) was found between TCC and CNC (*p* = 0.41). Only the *p*-value for the alignment group indicated a statistically significant difference (*p* = 0.014), confirmed by the mean difference, which suggests that the CNC group had a higher average score compared with the TCC group. For all other variables, no statistically significant differences were observed between the means (Table 5).

**Table 5:** Summary of AOFAS outcomes for TCC and CNC at follow-up for patients’ feet treated with coalition resection and valgus correction with SESA and statistical significance between CNC and TCC for AOFAS score with subgroups (for *p* < 0.05, 95% confidence interval)

Confidence interval 95%							
Group	Stats	df	*p*	Mean difference	SE difference	Lower bound	Upper bound
PAIN	110.5	29.0	0.623	2.99 × 10*−5*	1.5467	−2.07 × 10^−5^	4.46 × 10^−5^
FUNCTION	117.0	29.0	0.902	0.00	0.8523	−2.96 × 10^−5^	2.88 × 10^−5^
Activity limitations	112.5	29.0	0.366	0.00	0.1939	−8.92 × 10^−5^	0.00
Max walking distance	112.5	29.0	0.366	0.00	0.0646	0.00	5.94 × 10^−5^
Walking surfaces	113.0	29.0	0.616	−2.93 × 10^−5^	0.2186	−3.16 × 10^−5^	5.57 × 10^−5^
Gait abnormality	103.5	29.0	0.276	5.80 × 10^−5^	0.4877	−1.21 × 10^−5^	5.04 × 10^−5^
Sagittal motion	105.0	29.0	0.178	0.00	0.2812	−632 × 10^−5^	0.00
Hindfoot motion	119.0	29.0	0.973	7.03 × 10^−5^	0.3737	−9.32 × 10^−5^	1.59 × 10^−5^
ALIGNMENT	80.0	29.0	0.014*	6.97 × 10^−5^	0.6092	0.00	5.00
AOFAS TOTAL	100.5	29.0	0.418	3.48 × 10^−5^	2.0509	−1.94 × 10^−5^	7.00

*Statistically significant; independent samples tests (Mann–Whitney). *stats* statistics, *df* degree of freedom, *p* = *p*-value, *SE* standard error of the difference

## Discussion

Among tarsal coalitions, TCC and CNC are the most frequent, both leading to symptomatic rigid flatfoot deformity in most of the cases [6–8, 32]. Flatfoot deformity with an abnormally low or absent arch, as well as the valgus of the hindfoot with the rigid excessive eversion of the heel, are associated with coalitions in most of the cases. The hindfoot valgus at first evaluation in adolescents and the residual valgus deformity later in adult age have been addressed in many studies [16–21].

Pain represents a main symptom and a consensual indication for treatment [18], with resection as gold standard for persistently painful TCC [33, 34]. However, many authors reported poor surgery outcomes after coalition resection when hindfoot valgus deformity remains [17–19]. When untreated hindfoot valgus deformity is present, the lateral submalleolar region becomes painful due to calcaneal-malleolar impingement [15, 16]. According to literature data, the valgus deformity represents the main cause of pain in adult life. Mosca and Bevan reported that if there is excessive hindfoot valgus deformity, with or without a resectable coalition, a calcaneal lengthening osteotomy for valgus correction is necessary for pain relief [18]. Also considering CNC, if a valgus deformity is present, an Evans osteotomy or medializing calcaneal osteotomy is suggested in association to the bar resection [32]. Taking Achilles tendon tightness into account, Mosca et al. reported on the treatment of valgus deformity in 8 patients (13 talocalcaneal coalitions) using calcaneal lengthening osteotomy combined in all cases with concurrent gastrocnemius or Achilles tendon lengthening [18]. In contrast, in our recent study all 25 symptomatic patients (32 rigid flatfeet: TCC, *n* = 16; CNC, *n* = 16) underwent coalition resection and minimally invasive subtalar arthroereisis (SESA) in a one-step procedure to correct residual hindfoot valgus. Gastrocnemius or Achilles tendon lengthening was never required, likely because calcaneal lengthening osteotomy—and the consequent alteration of rearfoot anatomy—was avoided, thereby preserving the native insertion of the Achilles tendon. Moreover, in our cohort, stretching of the Achilles tendon was performed pre- and postoperatively when clinically indicated [23]. Furthermore, Zaidman et al. retrospectively reviewed cases of 10 children (11 feet) with symptomatic talocalcaneal tarsal who underwent calcaneal lengthening osteotomy and coalition resection (RC). On the basis of ankle dorsiflexion at the end of the procedure, the Achilles tendon was lengthened only in half of the cases, underlying that calcaneal lengthening was responsible for that indication. The same authors also reported concerns regarding possible inadvertent overlengthening and weakening [35].

Other studies reported the need for valgus deformity correction at the onset of foot pain or according to the dimensions of the coalition and the valgus severity. Osteotomies and lateral column lengthening are the surgical options for deformity correction or arthrodesis as salvage procedure. Realignment surgery performing bony procedures is the most common suggested strategy described in literature [17, 36–38].

**Fig. 5:**
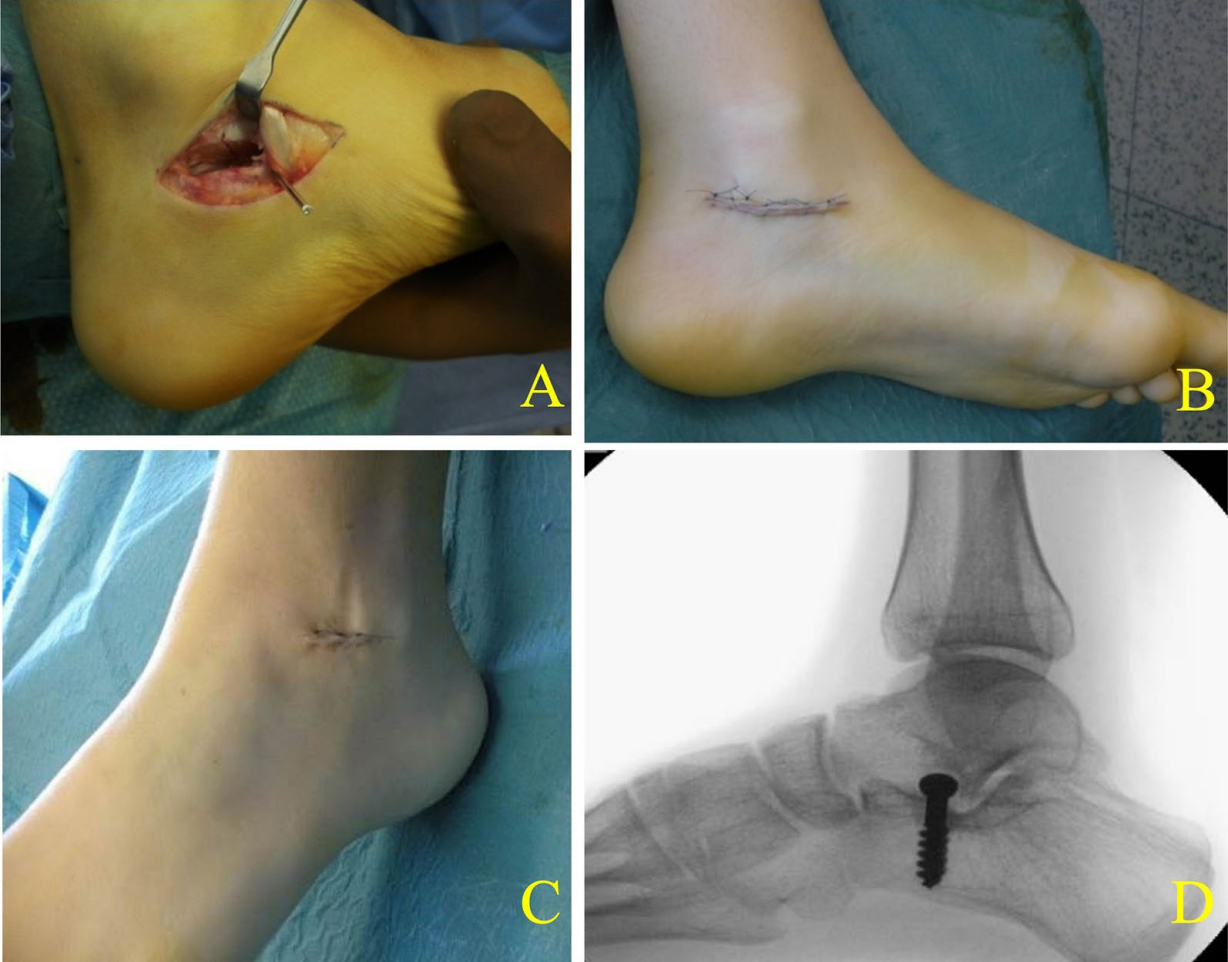
Minimally invasive procedure for TCC resection and hindfoot valgus deformity correction with SESA. **A** Resection side. **B** Medial approach incision after skin suture. **C** Lateral approach incision and skin suture after SESA. **D** Lateral radiographic view at FU before screw removal

In our study, patients underwent a minimally invasive procedure (SESA) for correction of the valgus deformity planning a surgical approach, which considers performing both resection and valgus deformity correction at adolescent age in one step before joint degenerations appear (Fig. 5).

According to the literature, the main indication for subtalar arthoereisis is surgical correction of symptomatic flexible flatfoot in children. SESA showed a lower percentage of complications (6.38%) as reported in a review regarding 1856 feet in comparison with other techniques of subtalar arthroereisis with position of the implants into the tarsal canal in 691 feet (9%) [27].

In addition, Zaret and Myerson previously reported advantages of technique that avoid placing the screw across the subtalar joint but into the calcaneus as in SESA [39].

Moreover, Yen-Douangmala et al. analyzed the role of subtalar arthroereisis in pediatric and adult populations, but reported data exclusively regarding subtalar arthroereisis into the tarsal canal [40].

SESA technique is widely used in Europe [27, 28, 41] and only more recently in North America [29], where more bony procedures were often suggested for flexible flatfoot deformity in children [40, 42–45]. Sullivan et al. reported overall patient satisfaction of 90% with the procedure [29]. Moreover, a comparison between SESA and standard technique of modified Evans calcaneal osteotomy was recently published by authors from the USA [46].

SESA was initially performed for flexible flatfoot at the age of 10.6 ± 1.9 years [47]. However, in younger patients, after initial correction a recurrence is described. Thus, the appropriate age for surgical indication has been postponed, and according to data published later, the mean age was 11.5 ± 1.81 years, with only occasional recurrence [26]. In the present study, age at surgery was 12.8 ± 1.4 years.

In tarsal coalition, flatfoot valgus deformity is not flexible. However, after resection of tarsal coalitions, when the foot becomes mobile and flexible again in the subtalar joint and a severe valgus deformity remains, arthroereisis can be indicated [48] and can follow coalition resection as a one-step procedure [23, 49].

Excellent radiological and functional results at median FU of 4.9 years and at a median patient age of 16.8 years are reported in a retrospective study of De Pellegrin et al. [23]. In this cohort the mean pre- and postoperative talo-calcaneal angle according to Costa-Bartani (normal values 120–125°) [50] for TCC were, respectively, 143.7° ± 7.7° and 129.7° ± 7.0° (*p* < 0.0001) and 141.5° ± 7.7° and 130.5° ± 5.2° (*p* < 0.0001) for CNC. Similarly, the mean talar inclination angle (normal range, 20–30°) improved from 31.2° ± 6.4° to 21.4° ± 3.4° for TCC (*p* < 0.0001) and from 29.2° ± 5.3° to 19.3° ± 1.6° for CNC (*p* < 0.0001).

Other authors reported few cases of resection and subtalar arthroereisis for valgus correction. Giannini et al. reported resection of TCC and use of a bioabsorbable screw as endorthesis in the subtalar joint for subtalar arthroereisis in 12 patients with excellent and good results in 8 (57.1%) [48]. Di Gennaro et al. [51] used in a non-bioabsorbable screw introduced into the talus with the aim of obtaining a “calcaneo-stop” effect in association to the TCC resection in 21 patients with statistically significant postoperative AOFAS score improvement. Knörr et al. reported 4 calcaneo-stop procedures simultaneously performed among 15 patients with symptomatic TCC after arthroscopic resection and a hindfoot valgus > 20°, with statistically significant increase of AOFAS score to 90.9 postoperatively [52].

In this retrospective study, functional outcomes after resection and SESA were evaluated at a mid-term average follow-up of 4.7 ± 3.2 years with a mean age of the patients at FU of 17.5 ± 3.1.

Overall results showed an excellent AOFAS average score of 95.6 ± 5.7 with no recurrence of the hindfoot valgus deformity.

Regarding pain and function, no statistical significance was present between TCC and CNC. Regarding alignment, better statistically significant outcomes could be evaluated in CNC (Table 5).

At FU, four feet, two TCC and two CNC, were rigid but not showing a recurrence of the hindfoot valgus deformity. These data suggest that the valgus deformity and not the coalition itself represents more probably the cause of pain.

There are many limitations in this study. Preoperative imaging was incomplete considering CT and MRI. However, when x-ray images clearly demonstrate coalition, indication for MRI was only considered, particularly in CNC, if doubtful or negative radiological x-ray report was present despite the clear findings at clinical examination with pain as main symptom. CT was more indicated in TCC for surgical planning.

Preoperative AOFAS was not available. However, AOFAS score is considered more a valid tool for evaluation of functional outcomes at FU than a method for comparison of pre- and postoperative function, which can be evaluated in coalitions also through classical parameters such as pain, range of motion, and hindfoot alignment.

Midterm and not long-term results are reported in this study. However, regarding SESA for valgus correction, long-term results are available in literature [27, 41]. This technique shows unquestionable efficacy, maintaining hindfoot alignment in flexible flatfeet even after later routinely removal of the screw [27].

In the present study, screw removal was not performed routinely. Only two patients underwent screw removal due to pain after 3 and 5.6 years, respectively, and five other patients underwent screw removal in asymptomatic feet after a mean time of 5.2 years (range 4–6 years).

Other limitations of this study were the lack of a patient satisfaction survey and radiological evaluation at follow-up. However, during the long follow-up period, nearly all patients were functionally satisfied with the clinical and functional outcomes.

In conclusion, symptomatic rigid flatfeet affected by CNC and TCC can be treated with coalition resection and a minimally invasive subtalar extraarticular screw arthroereisis (SESA) for correction of hindfoot valgus in one step at adolescent age.

According to our results, minimally invasive SESA is a valid, alternative procedure to calcaneal osteotomy and multiple osteotomies in adolescents to realign the hindfoot and to avoid arthrodesis in adulthood. The surgical options for painful flexible flatfeet described in literature were more complex procedures with multiple osteotomies and lengthy recovery.

## Data Availability

The datasets used and/or analyzed during the current study are available from the corresponding author on reasonable request.
